# Mental health of healthcare professionals during the early stage of the COVID-19 pandemic in Ethiopia

**DOI:** 10.1192/bjo.2020.130

**Published:** 2020-12-01

**Authors:** Yimenu Yitayih, Seblework Mekonen, Ahmed Zeynudin, Embialle Mengistie, Argaw Ambelu

**Affiliations:** Department of Psychiatry, Jimma University, Ethiopia; Department of Environmental Health Science and Technology, Jimma University, Ethiopia; Department of Medical Laboratory Sciences and Pathology, Jimma University, Ethiopia; Department of Environmental Health Science and Technology, Jimma University, Ethiopia; Department of Environmental Health Science and Technology, Jimma University, Ethiopia

**Keywords:** COVID-19, distress, health care professionals, insomnia, psychological

## Abstract

**Background:**

The coronavirus (COVID-19) pandemic causes healthcare professionals to suffer mental health problems such as psychological distress, anxiety, depression, denial and fear. However, studies are lacking related to Ethiopia and to Africa in general.

**Aims:**

To study the mental health of healthcare professionals during the COVID-19 pandemic in Ethiopia.

**Method:**

A hospital-based cross-sectional study was conducted at Jimma University Medical Center among 249 healthcare professionals. The data were collected using self-administered questionnaires between 22 and 28 March 2020. The psychological impact was assessed using the Impact of Event Scale – Revised (IES-R) and symptoms of insomnia were measured using the Insomnia Severity Index (ISI). Social support was evaluated using the three-item Oslo Social Support Scale. Data were analysed using logistic regression to examine mutually adjusted associations, expressed as adjusted odds ratios. The psychosocial status of the healthcare professionals was predicted using a classification tree model supported by the genetic search method.

**Results:**

The prevalence of psychological distress among healthcare professionals was 78.3%. The mean IES-R score was 34.2 (s.d. = 19.4). The ISI score indicated that the prevalence of insomnia was 50.2%. Higher psychological distress was associated with younger age, having insomnia, not having a daily update on COVID-19, and feeling stigmatised and rejected in the neighbourhood because of hospital work.

**Conclusions:**

This study indicates that, in Ethiopia, the prevalence of psychological distress among healthcare professionals is high and associated with specific sociodemographic risks.

Globally, control of infectious disease outbreaks continues to be a major health challenge.^1^ Cross-species transmission of animal and human viruses may allow exchange of genetic material and create a new virus with the possibility of bringing about a severe pandemic.^2^

COVID-19 is a global pandemic caused by severe acute respiratory syndrome coronavirus 2 (SARS-CoV-2), which is a beta-coronavirus that can be spread to humans through intermediate hosts such as bats.^3^ The leading cause of transmission is reported to be human to human via virus-laden respiratory droplets.^4^ Some healthcare professionals have low levels of knowledge about COVID-19, which might put them and their colleagues at risk of infection with SARS-CoV-2. Many patients with COVID-19 have atypical clinical manifestations and there is therefore the chance that they might be referred to several medical departments if practitioners do not recognise the disease.^5^ Patients may be infectious during the period of incubation, and that may place many healthcare professionals at risk of infection through contacts they make with patients. Research findings indicate that, in addition to droplet and contact transmission, SARS-CoV-2 might be transmitted by the faecal–oral route.^4^

Apart from the direct infection risks due to close contact with patients and potentially infectious co-workers during the COVID-19 pandemic, healthcare professionals are under increasing stress and mental health risks, as they were during the SARS epidemic.^6^ Different pieces of evidence indicate that healthcare professionals suffered psychological distress such as anxiety and depression at varying levels during the SARS outbreak in 2003.^7,8^ This distress is aggravated by severely inadequate personal protective equipment (PPE) in hospitals and worsened by the implementation of traffic control bundling. Although we accept that shortages of PPE have been much worse in low-income counties, they have also affected higher-income countries.^9^

Research on previous disease outbreaks has shown that many healthcare workers presented high levels of psychological distress, frequent concerns about their own and their families’ health, worries about their performance of daily activities, and fears of stigmatisation by local communities.^10–12^ During the previous SARS outbreak, worry and distress were associated with higher job stress, social isolation and health fears among healthcare professionals.^10,11^

The outbreak of COVID-19 in Ethiopia officially started on 13 March 2020, after a Japanese person arrived in Ethiopia from Burkina Faso and tested positive for the novel COVID-19. There was a subsequent surge of cases, with a peak of 124 new infections recorded on 27 April, by which time three deaths had occurred and several exposed healthcare workers were under quarantine because they had been in contact with patients. Such interactions lead to increased stress in the healthcare workforce, which could result in a serious weakening of the health service delivered.

There is no information available regarding the psychological impact of the COVID-19 pandemic on healthcare professionals in Ethiopia. Given the possibility of a future pandemic, more systematic research is needed to improve understanding of the psychological impacts of the COVID-19 pandemic and related risk and protective factors. To address knowledge gaps, this study describes the mental health status of healthcare professionals during the COVID-19 pandemic in Jimma University Medical Center (JUMC), Ethiopia.

## Method

We used a hospital-based cross-sectional study design. This study was conducted between 22 and 28 March 2020 at JUMC, which is the largest health facility in south-western Ethiopia, having 692 beds. JUMC provides referral medical services to patients coming from different health facilities in south-western Ethiopia. JUMC is leading most of the prevention, detection and patient care related to COVID-19 in the region. It is also strengthening its capacities and providing facilities for quarantine and treatment services.

At the time of the COVID-19 outbreak, 1256 health professionals were working in JUMC, 249 of whom were invited to participate in the present study. Data were collected from different departments of JUMC using questionnaires. Each of the data collectors had an MSc in a health-related field. Researchers supervised the data collection process. Investigators gave 1 day of training for the data collectors on the objectives of the study and how to approach and handle questions. Healthcare professionals were stratified on the basis of the type of profession (with four categories: doctor, nurse, pharmacist and laboratory technologist). The number of sample points was determined using a proportional allocation formula for each stratum. To select an individual health professional from each profession, a systematic sampling method was employed using hospital employee rosters. The first health worker was selected by a lottery method. Participants completed a self-report paper questionnaire with instructions to complete within 1 week.

The study was ethically approved by the institutional review board (IRB) of Jimma University (approval reference number IRB00097\20). Verbal informed consent was sought from every respondent after explaining the confidentiality of data that would be obtained from each study participant. Reasonable physical distance was kept between the involved individuals during data collection. The data were collected in private and kept confidential.

### Measurements

The questionnaire asked about sociodemographic characteristics, psychological distress, insomnia and social support. Demographic variables included age, gender, marital status, education, occupation and monthly income.

The Impact of Event Scale – Revised (IES-R)^13^ was used to measure the psychological response and determine post-traumatic stress symptoms experienced by participants during the week following the COVID-19 outbreak. Respondents were informed that the items constituted a list of ways they may have felt or behaved during that week, and that they should indicate the frequency of occurrence of each symptom on a four-point scale.

The IES-R has 22 items with a Likert rating scale ranging from 0 (not at all) to 4 (extremely), giving a total score ranging from 0 to 88. The scale has confirmed reliability and validity for measuring post-traumatic stress symptoms across diverse cultural settings. The IES-R is generally not used to diagnose post-traumatic stress disorder (PTSD) in clinical settings, but cut-off scores have been used for a preliminary diagnosis of PTSD and it is widely used for screening at-risk patients with post-traumatic stress.^14–16^ A total IES-R score ≥9 signifies the likely presence of psychological distress.^17^

Symptoms of insomnia were measured by the Insomnia Severity Index (ISI).^18^ This scale is widely used to assess the nature, severity and impact of insomnia. It consists of seven items scored on a 0–4 Likert scale and summed to give a total score that ranges from 0 to 28. The final ISI score was categorised into no insomnia (0–7 score), mild insomnia (8–14 score) and moderate to severe insomnia (15–28 score).^19^

The three-item Oslo Social Support Scale assesses level of social support.^20^ The sum of the scores on the three items ranges from 3 to 14, and total scores are divided into three broad categories: poor social support (a score of 3–8), moderate support (9–11) and good support (12–14). A reliability and validity assessment done in Nigeria yielded a Cronbach's alpha coefficient of 0.50, and concurrent validity was low but significant.^21^

### Statistical analysis

The data were extracted, edited and analysed using the Statistical Package for Social Sciences (SPSS) version 23 for Windows. Frequency tables were constructed to summarise the sociodemographic characteristics and prevalence of psychological distress. Bivariate logistic regression was performed separately for each independent variable. Independent variables with *P* < 0.25 were entered into the final model for multivariable analysis. Variables in the mutually adjusted multivariable model with a two-sided *P*-value <0.05 were considered statistically significant.

Classification tree predictions of psychosocial distress were made using Weka 3.8 for Windows. Weka is an open-source machine learning software developed at the University of Waikato that can be accessed through a graphical user interface and is highly useful for data mining and knowledge generation.^22^ Model building was performed using the J48 algorithm in Java programming language; this is thought to be the best machine learning algorithm for scrutinising data categorically and continuously in order to generate a reliable classification or decision tree. The use of these algorisms helped to develop a transparent and easily understandable decision tree model. Repeated randomisation of the instances was done to develop a consistent model.

The predicting variables of the classification tree model were selected by the wrapper subset/attribute evaluator using a genetic search algorithm. The wrapper method searches for an optimal feature subset of variables and the genetic algorithm searches for the appropriate attribute based on the theory of survival of the fittest.^23^ This algorithm reflects the process of natural selection, where the fittest variables are selected in order to produce a reliable and stable predicting model. The main features of a genetic algorithm are that it works with the coding of the parameter set, not the parameters themselves; that it initiates its search from a population of points, not a single point; that it uses pay-off information, not derivatives; and that it uses probabilistic transition rules, not deterministic ones.^24^ When the genetic algorithm finds the best predicting variable, the wrapper evaluator algorithm approves or disregards the variables/attributes involved in the model.

The model performance was evaluated based on the kappa statistic and the percentage of correctly classified instances (%CCI): κ > 0.2 and %CCI > 80% were considered a good model.

## Results

### Demographic characteristics

A total of 249 participants were recruited in this survey, with a response rate of 99.6%. Of this total, 131 (52.6%) were women; the mean age was 27.4 years (s.d. = 4.1; range 22–50 years). The highest proportion, 225 (90.4%), were from the age group 22–30 years old. In terms of profession, 130 (52.2%) were nurses, 86 (34.5%) doctors, 33 (13.2%) other hospital staff. Overall, the mean length of work experience of participants was 3.7 years (s.d. = 3.6). The majority of participants (138; 55.4%) were Orthodox Christian by religion. Of study participants, 151 (60.6%) were single (Table 1).

**Table 1:** Demographic characteristics of respondents (*n* = 249) stratified according to psychological distress

Variable	IES-R score ≥9	IES-R score ≤8	Total, *n*
Age group, years: *n* (%)			
22–30	178 (79.1)	47 (20.9)	225
31–40	15 (78.9)	4 (21.1)	
41–50	2 (40.0)	3 (60.0)	
Age, years: mean, s.d.	27.2 (3.5)	28.1 (5.6)	
Gender, *n* (%)			
Male	84 (71.2)	34 (28.8)	118
Female	111 (84.7)	20 (15.3)	131
Marital status, *n* (%)			
Single	113 (74.8)	38 (25.2)	151
Married	82 (84.5)	15 (15.5)	97
Divorced	0 (0.0)	1 (100.0)	1
Occupation, *n* (%)			
Doctor	62 (72.1)	24 (27.9)	86
Nurse	105 (80.8)	25 (19.2)	130
Pharmacy professional	12 (80.0)	3 (20.0)	15
Laboratory professional	16 (88.9)	2 (11.1)	18
Years of service experience, *n* (%)			
≤2	94 (75.8)	30 (24.2)	124
3–4	43 (79.6)	11 (20.4)	54
5–6	33 (91.7)	3 (8.3)	36
≥7	25 (71.4)	10 (28.6)	35
Sleep pattern, *n* (%)^a^			
No insomnia	75 (60.5)	49 (39.5)	124
Mild insomnia	70 (93.3)	5 (6.7)	75
Moderate to severe insomnia	50 (100.0)	0 (0.0)	50
Social support, *n* (%)^b^			
Poor social support	108 (78.8)	29 (21.2)	137
Moderate social support	69 (83.1)	14 (16.9)	83
Good social support	18 (62.1)	11 (37.9)	29

IES-R, Impact of Event Scale – Revised.

a. Symptoms of insomnia were measured using the Insomnia Severity Index.

b. Social support was evaluated using the three-item Oslo Social Support Scale.

### The prevalence of psychological distress

The prevalence of psychological distress based on an IES-R score ≥9 was 195 (78.3%). The mean IES-R score was 34.2 (s.d. = 19.4). Of all respondents, 22 (8.8%) received a score in the range 9–25, indicating the presence of mild psychological distress; 101 (40.6%) scored 26–43, indicating moderate psychological distress; and 72 (28.9%) exceeded the cut-off score of 46, indicating severe psychological distress. The mean IES-R Intrusion score was 13.7 (s.d. = 6.9), and the mean Avoidance and Hyperarousal scores were 12.3 (s.d. = 6.8) and 9.2 (s.d. = 5.8).

A higher prevalence of psychological distress was seen among nurses (53.8%) and doctors (31.8%) compared with laboratory professionals (8.2%) and pharmacy professionals (6.2%). Among those who had psychological distress, the prevalence was highest among females (56.9%) compared with males (43.1%). A higher prevalence of psychological distress was seen among single (57.9%) compared with married people (42.1%). In terms of length of work experience, prevalence of psychological distress was highest (48.2%) among respondents who had ≤2 years of experience, compared with 3–4 years (22.1%), 5–6 years (16.9%) and ≥7 years (12.8%).

### Insomnia, social support and psychosocial distress

Of the 249 participants, 50.2% met the criteria for insomnia based on an ISI score ≥8. Respectively, 49.8, 30.1 and 20.1% of participants reported no insomnia, mild insomnia and moderate to severe insomnia. The prevalence of insomnia was highest among nurses (59.2%) and doctors (24.8%), compared with pharmacy professionals (5.6%). The prevalence of psychological distress was also higher among respondents who had insomnia (61.5%) compared with those with no insomnia (38.5%).

As regards social support, 55.4% of participants had poor social support (a score of 3–8 on the Oslo Social Support Scale), 35.4% had moderate social support (a score of 9–11) and 9.2% had good social support (a score of 12–14).

**Fig. 1:**
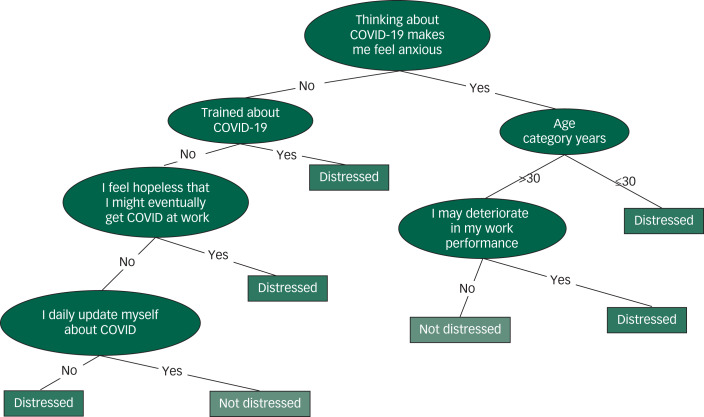
The decision tree model identifying personal factors affecting the psychosocial status of healthcare professionals at Jimma University Medical Center (κ = 0.31, percentage of correctly classified instances %CCI = 81.93).

The classification tree model revealed that the health workers’ psychosocial distress was mainly influenced by their anxious feelings when thinking about COVID-19, being young (<30 years of age), feeling hopeless about the probability of contracting COVID-19 infection at work, lack of training about COVID-19, fear of deterioration in work performance and not obtaining a daily update about the virus (Fig. 1).

### Factors associated with psychological distress

Table 2 shows the distribution of factors that were associated with psychological distress among the respondents. We conducted bivariate logistic regression analyses of all 14 factors. Among the 14 input variables considered in the bivariate analysis, 12 were nominated for multivariable analysis (*P* < 0.25): age; gender; marital status; occupation; years of service; social support; insomnia; an update about COVID-19 information daily; feeling that my family will not look me if I was infected; feeling that my institution didn't support me during the COVID crisis; feeling stigmatisation and rejection by the neighbourhood; and satisfaction with government handling of COVID.

**Table 2:** Bivariate analysis for psychological distress (*n* = 249)

Variable	IES-R score ≥9, *n* (%)	IES-R score ≤8, *n* (%)	COR	95% CI	*P*
Age group, years					
22–30	178 (79.1)	47 (20.9)	5.7	0.9–35.0	0.061*
31–40	15 (78.9)	4 (21.1)	5.6	0.7–46.0	0.107*
41–50	2 (40.0)	3 (60.0)	1		
Gender					
Male	84 (71.2)	34 (28.8)	1		
Female	111 (84.7)	20 (15.3)	2.2	1.2–4.2	0.011*
Marital status					
Single	113 (74.8)	38 (25.2)	0.6	0.3–1.1	0.101*
Married + divorced	82 (83.7)	16 (16.3)	1		
Occupation					
Doctor	62 (72.1)	24 (27.9)	0.3	0.1–1.5	0.151*
Nurse	105 (80.8)	25 (19.2)	0.5	0.1–2.4	0.410
Pharmacy professionals	12 (80.0)	3 (20.0)	0.5	0.1–3.5	0.484
Laboratory professionals	16 (88.9)	2 (11.1)	1		
Years of service experience					
≤2	94 (75.8)	30 (24.2)	1.3	0.5–2.9	0.599
3–4	43 (79.6)	11 (20.4)	1.6	0.6–4.2	0.375
5–6	33 (91.7)	3 (8.3)	4.4	1.1–17.7	0.037*
≥7	25 (71.4)	10 (28.6)	1		
Insomnia					
No	75 (60.5)	49 (39.5)	1		
Yes	120 (96.0)	5 (4.0)	15.7	6.0–41.1	<0.001*
Social support^a^					
Poor social support	108 (78.8)	29 (21.2)	2.3	0.9–5.3	0.059*
Moderate social support	69 (83.1)	14 (16.9)	3.0	1.2–7.7	0.022*
Good social support	18 (62.1)	11 (37.9)	1		
Having update on COVID daily					
Yes	117 (74.1)	41 (25.9)	1		
No	78 (85.7)	13 (14.3)	2.0	1.0–4.0	0.046*
I don't feel confident an employee will care for me if I get COVID-19					
Yes	120 (80.0)	30 (20.0)	1.3	0.7–2.4	0.427
No	75 (75.8)	24 (24.2)	1		
I feel I will transmit COVID-19 to my family					
Yes	137 (79.2)	36 (20.8)	1.2	0.6–2.2	0.612
No	58 (76.3)	18 (23.7)	1		
I feel my family will not look after me if I was infected					
Yes	78 (85.7)	13 (14.3)	2.1	1.1–4.2	0.034*
No	117 (74.1)	41 (25.9)	1		
I feel that my institution did not support me during the COVID crisis					
Yes	137 (82.5)	29 (17.5)	2.0	1.1–3.8	0.024*
No	58 (69.9)	25 (30.1)	1		
Overall satisfied with government handling of COVID in the community					
Yes	83 (78.3)	23 (21.7)	1		
No	112 (78.3)	31 (21.7)	1.0	0.5–1.8	0.997
Feeling stigmatisation and rejection in the neighbourhood because of hospital work					
Yes	122 (87.1)	18 (12.9)	3.3	1.8–6.3	0.001*
No	73 (67.0)	36 (33.0)	1		

IES-R, Impact of Event Scale – Revised; COR, crude odds ratio.

a. Social support was evaluated using the three-item Oslo Social Support Scale.

* *P* < 0.25.

### Multivariable logistic regression analysis

We used stepwise forward selection strategies to select variables for the multivariable prediction of psychological distress. The multivariable model revealed that psychological distress increased with being young, having insomnia, not having an update on COVID daily and feeling stigmatisation and rejection by the neighbourhood because of hospital work (Table 3).

**Table 3:** Results of multivariable logistic regression analysis to examine factors associated with psychological distress based the Impact of Event Scale – Revised score

Independent variable	Adjusted OR (95% CI)	*P*
Age group, years		
22–30	24.5 (1.2–492.6)	0.037*
31–40	13.9 (0.6–307.7)	0.095
41–50	1	
Insomnia		
No	1	
Yes	19.2 (6.0–61.5)	<0.001*
Having update on COVID daily		
Yes	1	
No	2.6 (1.0–6.6)	0.042*
Feeling stigmatisation and rejection in the neighbourhood because of hospital work		
Yes	2.7 (1.1–6.4)	0.025*
No	1	

OR, odds ratio.

* *P* < 0.05.

## Discussion

This study is the first of its kind investigating the impact of the COVID-19 pandemic on the mental health status of healthcare professionals in Ethiopia. The findings reveal that the prevalence of psychological distress based on an IES-R score ≥9 was 78.3%. The study revealed that the majority of younger health workers suffered psychosocial distress. This was been confirmed by a classification tree model. Participants with insomnia, those without up-to-date information about COVID-19 on a daily basis, and those who feared stigmatisation and rejection by their neighbourhood were psychologically distressed.

The overall prevalence of psychological distress in this study, at 78.3%, was higher than the rate reported in China (71.5%).^25^ This is probably due to a lower mental preparedness, less rigorous infection control measures, poor psychosocial support system, limited capacity of healthcare institutions and inadequate availability of PPE in Ethiopia after the COVID-pandemic was declared. Furthermore, healthcare professionals were working in close contact with people at high risk of being infected with COVID-19 and are highly exposed to the hazard of contracting the disease from their patients.^26^ Healthcare professionals were distressed not only because they feared infection but also because the increased number of patients with COVID-19-related problems intensified their case-loads and increased their working hours.^27^ These findings highlight the value of giving psychological support to reduce the remarkable stress during the COVID-19 pandemic.

The prevalence of insomnia was 50.2% among healthcare workers during this COVID-19 pandemic, higher than in Wuhan during the COVID-19 pandemic (34%) and in Taiwan (37%) during the SARS epidemic.^25,28^ Having insomnia was found to be a significant independent predictor of psychological distress. Sleep disorders lead to activation of the hypothalamic–pituitary–adrenal (HPA) system, thereby leading to increased distress.^29^ Stress involves increased psychological and physical activation in response to demand, thereby promoting a vicious cycle of stress and insomnia.^29^ The Taiwan research^28^ revealed that during the SARS outbreak, sleep quality among medical staff was poor at the start of the crisis and progressively improved after 2 weeks, suggesting that insomnia was related to contagion outbreak-induced stress. The primary source of stress among healthcare workers in the present study was related to the highly contagious nature of COVID-19.

The multivariable model revealed that psychological distress increased with younger age. This is consistent with a study conducted in Australia during an influenza epidemic.^30^ The possible reason may be that younger people were most at risk and were coping less well with the consequences. Studies indicate that younger people are less resilient or skillful in the handling of difficult situations, such as the COVID-19 pandemic.^31^

Healthcare professionals who did not receive a daily update about COVID-19 had more risk of psychological distress compared with those who received daily updates. Those who updated daily became more confident about infection control, possibly diminishing the collective sense of threat, which has a protective function and minimises distress levels. It is speculated that healthcare workers could benefit from receiving daily information about COVID-19 and even rehearsals for future pandemics.

Those feeling stigmatisation and rejection in the neighbourhood because of hospital work were at higher risk of developing psychological distress compared with their colleagues. These results are congruent with reports from earlier studies that perceived stigma and feelings of rejection were a significant predictor for psychological distress.^32^ Fear and anxiety about the disease in the community can lead to social stigma towards healthcare workers.^33^ Communities associate COVID-19 infection with healthcare workers, even though not every health worker is at risk for the disease.

These results recommend that there is a role for providing truthful and well-timed COVID-19 facts to healthcare workers and the community to reduce ambiguity and decrease stigmatisation of healthcare workers. Providing appropriate accommodation for healthcare workers might be helpful for those who are worried about the danger of infecting their families.

### Clinical implications

Healthcare professionals engaged in treating patients with highly infectious diseases are likely to have experience of unpredictable and uncertain psychological distress. Thus stress relief activities such as physical exercise, peer support, yoga, meditation, or religious or spiritual practices might act as early and prompt prevention.^34^ Addressing mental health problems in medical workers is thus essential for the better prevention and control of the COVID-19 pandemic.^35^ Healthcare workers usually provide care for confirmed or suspected cases, which makes them more distressed in the absence of personal protective equipment (PPE). Hence, by strengthening PPE supplies, it is possible to ease the pressure on healthcare personnel. Furthermore, online and electronic media broadcasts on reducing the risk of transmission between patients and medical workers in medical settings could reduce the pressure on medical workers.

A culturally appropriate psychological crisis intervention plan should be developed, and training for healthcare workers on awareness and how to reduce the psychological impact of COVID-19-induced distress should be provided. Promoting the psychological well-being of healthcare workers and providing psychosocial support will also mitigate the psychological effects of the COVID-19 pandemic.^36^ It would therefore be timely for actors in the Ethiopian public healthcare system to introduce novel approaches to generate financially sustainable programmes to prevent psychological distress among healthcare workers through a group of well-trained psychologists. Furthermore, the training provided to health professionals needs to be monitored as it must fit in with already busy clinical schedules.

### Limitations

Our study has limitations. We collected the data for the week after the COVID-19 outbreak in Ethiopia. Consequently, the period of exposure to the pandemic had been short and we could only study the acute psychological impact, so our findings might not be generalisable to subacute and long-term psychological complications if the outbreak continues. Another limitation is that participants might have given socially desirable responses. Also, this was a cross-sectional study not able to determine cause-and-effect relationships between insomnia and psychological distress. Finally, longitudinal studies might help to assess for development or even a potential rebound effect of psychological distress once the impending threat of COVID-19 resolves.

## Data Availability

The data-sets generated and analysed during the study presented in this paper are part of an ongoing project and we will make them available to organisations and individuals on the basis of official requests.
